# Balancing on a slack line – Staffs’ experiences of talking about sexuality and sexual health with patients cared for in forensic psychiatry in Sweden

**DOI:** 10.3389/fpsyt.2024.1450377

**Published:** 2024-09-03

**Authors:** Anna Lindskog, Malin Lindroth, Kristina Holmgren, Anna Birgitta Gunnarsson

**Affiliations:** ^1^ Department of Health and Rehabilitation, Institute of Neuroscience and Physiology, Sahlgrenska Academy, University of Gothenburg, Gothenburg, Sweden; ^2^ Department of Research and Development, Region Kronoberg, Växjö, Sweden; ^3^ Faculty of Health Sciences, Department of Behavioural Science, Oslo Metropolitan university, Oslo, Norway

**Keywords:** healthcare professionals, interview, mental health, qualitative content analysis, sexual health, forensic psychiatry care

## Abstract

Sexuality is a central part of being human, however, talking about sexual health is generally avoided in forensic psychiatry. The aim of this qualitative study was to explore how healthcare professionals experience talking about sexuality and sexual health with patients cared for in forensic psychiatry. Individual interviews were conducted with eighteen healthcare professionals from ten different forensic psychiatry care units in Sweden. The interviews were semi-structured, and the data was analyzed with qualitative content analysis. The findings showed the overarching theme “Balancing on a slack line”. The conversations the healthcare professionals have about sexuality and sexual health with patients in this setting are affected by forensic psychiatry’s dual mission; to provide care and at the same time protect patients and society. These conversations can be about opening up for having responsive conversations, but also closing conversations since the field of sexuality and sexual health is fraught with norms and preconceptions. To be able to conduct these conversations can be a challenge since the professionals can experience uncertainty due to a lack of competence, indicating that knowledge and resources are needed to facilitate conversations. In conclusion, the study indicates that there is a lack of support and structure for performing conversations about sexuality and sexual health in forensic psychiatry care, and there is a need for increased knowledge among healthcare professionals. In order to support staff, the forensic psychiatry services need to acknowledge the national guidelines for sexual and reproductive health and rights and to develop and adapt the local guidelines.

## 1 Introduction

This study focuses on sexuality and sexual health for patients cared for in forensic psychiatry, and the healthcare professionals’ experiences of having conversations about this. Sexuality is a core part of being human throughout the lifespan encompassing e.g. sex, gender identities, sexual orientation, roles and relationships (1). Sexual health is part of a broader concept, termed sexual and reproductive health and rights (SRHR). In accordance with the Guttmacher-Lancet Commission’s definition of SRHR, everyone has the right to make decisions about their own body and to be given healthcare and other health promotion initiatives that support that right (2, 3). A Swedish strategy for work on SRHR was presented in 2020 and the overall goal is a good and equal sexual and reproductive health free from discrimination for the entire population (3). This thus applies to patients cared for within forensic psychiatry. Sexual concerns, together with other health needs, should be addressed since people continue to be sexual beings during illness and disabilities (4). Thus, in order to be able to achieve a good sexual health for patients, healthcare professionals who work close to the patients have to be attentive and competent when meeting their needs of support.

Offenders who have severe mental illness are treated according to special regulations in the Swedish legal system, and the conception of accountability is applied (5). They are, due to their severe mental illness, transferred to forensic psychiatric care instead of being in prison (5, 6). Patients in forensic psychiatry in Sweden are thus individuals who have been convicted of criminal acts (6). There were approximately 2070 patients who were cared for in forensic psychiatric care in Sweden in 2023, 85 percent of whom are men, and the median age is 40 years for women and 39 years for men (7). A large proportion of these convicted patients have committed violent crimes (8). Forensic psychiatric care in Sweden is involuntary, and the duration is not pre-determined, regardless of the type of criminal act. The caregiver’s mission is complex (5, 8), with the dual aims of providing mental healthcare to the patient, and at the same time protecting society from those patients who are at risk of recidivism (8, 9).

Most patients with severe mental illness in forensic psychiatry suffer from psychotic disorders, and it is also common with comorbidities such as neurodevelopmental disorders, personality disorders and substance abuse (8, 10). According to the Swedish National Forensic Psychiatric Register (7), the most common main diagnosis attributed to approximately half of the patients (51 percent of men and 45 percent of the women) is schizophrenia. The second most common main diagnosis is neuropsychiatric diagnosis, attributed to 11 percent of the men and 13 percent of the women (7). The third most common main diagnosis is personality disorders, attributed to 4 percent of the men and 7 percent of the women (7). The most common (51 percent among men and 45 percent among women) index crime, i.e. the criminal acts that led to forensic psychiatry care, are crimes against life and health such as murder, manslaughter and assault (7). The most common index crime for men and women with ongoing forensic psychiatric care in 2023 was assault (73 percent of men and 82 percent of women) (7). Seven percent (n=144) of the men had index crimes related to sexual assaults, while this type of crime was less than one percent among the women (7). This percentage shows that sex offenders are more often sentenced to prison than forensic psychiatry care in Sweden. Ten percent (n=711) of the prison population of just under 7000 in Sweden have been admitted to prison of sexual assault in 2023 (11).

Patients in forensic psychiatry do not only need medical treatment, rehabilitation and adaptation to society as well as the reduction of the risk of relapse are important elements in the provision of their care (8). Patients also need psychological and psychosocial interventions to enable rehabilitation and re-integration into society, and the importance of competent healthcare professionals has been highlighted for the facilitation of these encounters (9). The professionals should be available and show respect, alleviate the patients’ vulnerability and enable their recovery towards increased well-being and health (9).

Research focusing on sexuality and sexual health related to severe mental illness is still scarce, not least where patients in forensic psychiatry are concerned. There are, however, studies that emphasize that sexuality and sexual health should be addressed in patients’ care in forensic psychiatry (12–14). One reason for this conclusion is that patients are cared for in forensic psychiatry for long periods, and they spend their sexual and reproductive lives within that context, which in turn can entail that they have difficulties in initiating or sustaining relationships (13–16). Moreover, patients in forensic psychiatry can experience various negative sexual outcomes such as sexually transmitted infections (STIs) and unplanned pregnancies, and some patients are excessively vulnerable for negative sexual health outcomes due to their mental illness and therefore need sexual health promotion and support (16).

Furthermore, as stated above, a large proportion of patients within forensic psychiatry are diagnosed with schizophrenia and according to Boer et al. (17), the most prominent cause of sexual problems may be the use of antipsychotic drugs. However, there are several other factors than just medication that also influence sexual functioning, e.g. comorbid somatic disorders, relationship factors, previous sexual experiences (17). Psychiatric symptoms (e.g. negative symptoms) and institutionalization can affect the patients’ sexuality, and those suffering from schizophrenia thus need to be offered psychoeducation and relationship counseling (17). There is a risk of non-compliance with antipsychotic drugs when a patient attributes sexual dysfunction solely to side effects from medication and it is therefore important to discuss issues of sexuality with patients (17). Addressing these issues might improve the outcomes of rehabilitation (12). Patients in forensic psychiatric care need sexual health education, and healthcare professionals should provide this support, as well as routine medication reviews (14, 16, 18).

Studies using qualitative research have shown that conversations about sexual health are commonly avoided, and that issues of sexuality are not sufficiently raised with patients in forensic psychiatry (12, 19). To our knowledge, little is known about how healthcare professionals experience addressing sexuality with patients in forensic psychiatry. The aim of this study was thus to explore how healthcare professionals experience talking about sexuality and sexual health with patients cared for in forensic psychiatry.

## 2 Material and methods

### 2.1 Design

The study had an explorative qualitative design. Individual interviews with healthcare professionals were conducted to gain an in-depth understanding of the participants contextual experiences (20). A conventional qualitative content analysis with an inductive approach, according to Graneheim and Lundman (21, 22) was applied to capture these experiences in an adequate fashion.

The study was approved by the Swedish Ethical Review Authority (No 2021-01971) and conducted according to the principles of the Declaration of Helsinki (23). The participants received written and oral information about the study, and they provided their written consent to participate. The right to withdraw at any time and without explanation was emphasized, the participants were informed that the data they provided would remain confidential, and the results would be presented on a group level.

### 2.2 Setting and participants

The study focused on healthcare professionals working in forensic psychiatry care units in Sweden. There are 26 such units in the country, which are administered by the regional healthcare services (7). There are three different security levels; level 1 (very high security that can withstand extraction attempts and qualified escape attempts); level 2 (high security protection that can withstand escape attempts), and level 3 (tracking of the patients’ location) (6). The level on which each patient is to be cared for is determined by an assessment of four aspects of risks: risk of extraction, escape risk, risk of harming oneself or someone else (6).

Twenty head managers from forensic psychiatry care units receiving patients in Sweden were initially contacted by email, in which information about the study was provided. Thirteen of these agreed to spread information about the study within their care units. Two declined to participate (one due to lack of interest and the other to prevailing workloads at the clinic during the pandemic), and the remaining five never responded. The inclusion criteria for participation were healthcare professionals who worked close to patients within forensic psychiatric clinics, while the exclusion criteria were healthcare professionals who had been working less than six months in forensic psychiatric care units.

Thirty-two healthcare professionals contacted the first author via e-mail and declared their interest in participating. A purposeful sampling procedure was then applied, and potential participants were requested, via e-mail, to provide information about their profession, age, gender and how long they had worked in forensic psychiatry. Based on this information, the purposeful sampling proceeded. Since the aim of the study was to explore a variation of experiences among different professionals, a selection based on profession was first made, and secondly an even distribution related to age, gender and how long they had worked in forensic psychiatry was sought. A total of eighteen participants from ten different forensic care units in Sweden were interviewed. Three of the ten forensic psychiatry care units were so called regional clinics (of which there are five in Sweden, and which can receive forensic psychiatry patients from all over Sweden). The remaining seven were secure county units providing forensic psychiatry care for patients in their own county. All levels of security were represented in the responding units: the three units had all levels of security; one unit had security levels 1 and 2; four units solely had security level 2; four units had security levels 2 and 3, and one unit solely had security level 3. The ten forensic psychiatry care units were spread over nine different counties in Sweden. The sample thus covers a wide range of aspects of forensic psychiatric care in Sweden, see **Table 1**.

**Table 1:** Characteristics of the participants (*n* = 18).

Profession	
Assistant mental health nurse	4
Occupational therapist	3
Physiotherapist	1
Psychiatrist	2
Psychologist	2
Registered nurse/registered mental health nurse	3
Social pedagogue	1
Social worker	2
Gender	
Women/men	15/3
Age (years)	
Median (min-max)	40 (27-62)
Work experience within forensic psychiatry (years)	
Median (min-max)	5 (1-23)

### 2.3 Procedure

The interviews, the majority of which were conducted by the first author (AL), took place between October 2021 and March 2022. The second author (ML) and the last author (ABG) conducted two interviews each. The participants chose where they wanted to perform the interview and most of them chose a digital platform (Zoom or Teams), while two chose “face-to-face” interviews. Semi-structured interviews with open-ended questions, based on an interview guide developed for the study, were performed. The interview guide dealt with the participants’ experiences of talking about sexuality and sexual health with patients cared for in forensic psychiatry and covered five areas: 1) challenges or opportunities around having these conversations within forensic psychiatry, 2) skills and knowledge, 3) potential benefit or harm for patients to talk about sexuality and sexual health, 4) experiences of working with issues related to sexuality and sexual health and 5) potential improvement of the work. All interviews were conducted in Swedish. All the interviews were audio-recorded and transcribed verbatim, the duration ranged between 25 and 62 minutes (Median 35 minutes).

### 2.4 Data analysis

The data was analyzed with qualitative content analysis, using an inductive approach (21, 22). The qualitative content analysis, in accordance with Graneheim and Lundman (21, 22) was conducted in the following steps: 1) transcripts were read repeatedly to gain a sense of the whole; 2) parts of the transcripts were sorted into content areas; 3) content areas were divided into meaning units; 4) meaning units were condensed and labelled with a code; 5) various codes were compared based on differences and similarities; 6) codes were sorted into subcategories and categories that represented the manifest content; and 7) categories were compared and their underlying meaning, the latent content, were formulated into a theme. Both manifest and latent content analysis require interpretation (22), and the interpretations varied in depth and level of abstraction. The initial focus was on the manifest content of the interview transcripts since the aim of this study was to explore participants’ experiences. An analysis of the latent content was also applied resulting in one overarching theme that encompassed three categories and six sub-categories.

All the authors initially read through data separately to gain an overall understanding, and then identified meaning units from two interviews, and condensed data into codes. Thereafter, the first author (AL) continued the analysis, and the data was sorted into content areas and divided into meaning units with the use of MAXQDA, a software program for qualitative data analysis (24). To gain an overview, the analysis continued in a Word-document; condensing meaning units and labelling them with a code that formed a basis for iterative discussions between all the authors. The different codes were compared based on their differences and similarities, and then sorted into subcategories and categories; first by the first author (AL) and then with the last author (ABG). Finally, the categories were discussed together and formulated into a theme, first by AL and ABG, and then together with all the authors, who then carried out the analysis as a repeated process, until consensus was reached. The data analysis was carried out in Swedish. This manuscript was written in English and was edited by a native English-speaking reviewer who was also fluent in Swedish. The quotes in Swedish were present in the text, and the reviewer could double-check the accuracy of the translation in this review.

The researchers’ pre-understanding is an important aspect that needs to be taken into consideration. All of the authors are women. The first author (AL) is an occupational therapist with a Master of Science in sexology with several years of experience from forensic psychiatry. The other authors are all experienced in conducting qualitative research, from the mental health field as occupational therapists (ABG, KH) and as a school health nurse from the field of state care for young people (ML). These professional and scientific perspectives and experiences were useful throughout the analytic process.

## 3 Results

The results consist of one overarching theme that encompasses three categories, and six sub-categories, see **Table 2**. The first category shows that conversations about sexuality and sexual health with patients cared for in forensic psychiatry are affected by forensic psychiatry’s dual mission; to provide care and support the patients while also limiting their risk of exposure to and protecting society. The second category describes how these conversations can concern opening up and taking initiatives for having responsive conversations, but also closing conversations since the field of sexuality and sexual health is fraught with norms and preconceptions. The third category highlights that being able to conduct these conversations can be challenging since the professionals can experience uncertainty due to a lack of competence, indicating that knowledge and resources are needed to facilitate conversations. The three categories together form the overarching theme that illustrates how having conversations with patients in forensic psychiatry about sexuality and sexual health can be experienced by professionals as “Balancing on a slack line”.

**Table 2:** Illustration of theme, categories, and sub-categories, within the overarching theme “Balancing on a slack line” that describes professionals’ experiences of talking about sexuality and sexual health with patients cared for in forensic psychiatry.

Theme	Balancing on a slack line		
**Category**	Having a mission to provide care, but also to protect	Opening up for, but also closing conversations	Being able, but also lacking knowledge and resources
**Sub-category**	Supporting the patients	Taking initiatives for responsive conversations	Lacking competence creates uncertainty
	Limiting risks of exposure and protecting society	Dealing with norms and preconceptions	Having knowledge and tools facilitates conversations

### 3.1 Having a mission to provide care, but also to protect

Talking about sexuality and sexual health with patients is experienced as being affected by the dual mission of forensic psychiatry: to provide care and to protect. Participants underline that, in this work, it is important to depart from the single patients’ rehabilitative needs while limiting their risk for sexual exposure.

#### 3.1.1 Supporting the patients

The participants describe that their conversations concerning sexuality have been about promoting the patients’ development and recovery. Having carried out these conversations is seen as beneficial as the participants have gained an understanding of the patients’ needs and knowledge gaps, because they experience patients as having;

Extremely poor knowledge about sexuality, i.e. either culturally or for various reasons, intellectual disability, or they have never tried. So, they are often quite ill-equipped for a functioning sexual life, and I think that is also a concern for the future (P3).

Patients’ knowledge gaps are described as a lack of factual knowledge, for instance in terms of how the body works or sexually transmitted infections, and as a fear of using medication (contraceptives and psychotropic drugs). Patients are also described as lacking the emotional knowledge needed to express emotions and understand consensual and equal relationships.

The participants say that patients need individual person-centered support to master social skills and develop social relationships. This includes taking the patient’s background and current status into consideration, and sometimes using adapted conversation tools and approaches (e.g., pictures, easy language). Support is said to be more important when patients are approaching the end of their care and a return to society, and support needs are commonly described as concerning patients’ intimate relations and the side effects of medication. Taking responsibility and having these conversations about sexual health as part of general health, and taking patients’ thoughts seriously, is seen as risk reducing in terms of both compliance to treatment and potential relapse:

This is something we have to work with, it’s like our responsibility to help in some way. But if you have more of this holistic view of the human being, then it’s part of the whole, and then perhaps more is included for some individuals and less for others. And then it must be something you have to take responsibility for (P18).

#### 3.1.2 Limiting risks of exposure and protecting society

At the same time as the participants provide support, they also need to limit and protect patients to reduce their risk of exposure. This can include limiting unwanted sexual behavior, e.g., hindering a patient from being naked or masturbating in public on the ward, but also limiting the risk for transactional sex. These protective measures are described as motives for the “threshold ban”, where the patient’s room is solely for that patient

*You don’t touch each other, it’s quite, you can’t do that, not between patients, it’s stated. Yes, we have a threshold ban, you’re not allowed to be in someone else’s room* (P1).

As well as not being allowed in each other’s rooms, sexual relationships between patients are also forbidden. The participants describe this as a dissuasion from, or a prohibition of sexual relations between patients. Despite this, intimate relationships have occurred, and patients have e.g., become pregnant.

Limiting patients to reduce the risk of exposure to other patients is particularly referred to in relation to female patients and patients with gender dysphoria. The latter group are described as often having autism and other general difficulties in social interactions, which makes them even more vulnerable. The participants further describe occurrences of staff preventing patients’ choices of gender-crossing clothing and putting on make-up. Some staff have also refused to use the pronoun that a patient preferred. That female patients need to be limited and protected is described and motivated:

Most have had bad experiences from sexual contacts, everything from being exposed to things and, or having promiscuous behavior, that you have seen the possibility of, well, everything from getting hold of cigarettes/…/having committed sexual acts with male patients to get cigarettes or get a little money (P2).

The participants described that when a patient applies for privileges, such as a leave, discussions take place about which limitations and restrictions that should be applied. In the case of a patient convicted of a sexual offence, the staff say they need to be even more restrictive about what a patient is allowed to do on their own. The mission to protect (the society) is often prioritized higher than patients’ individual needs and rights for care:

Society, what we do to protect society is seen to be so very important. Sometimes, I think we, we sometimes abandon our patients, thanks to this protection that we somehow assume we are responsible for. And then it becomes more monitoring than nursing (P15).

### 3.2 Opening up for, but also closing conversations

It emerges in the second category that the participants need to be responsive in conversations with patients and focus on health promotion. They consider sexual health to be a part of general health and well-being, and thus that staff in forensic psychiatric care need to take responsibility and open up for conversations about sexuality and sexual health. However, this is seen as challenging, as conversations about sexuality and sexual health are also experienced as something difficult and taboo. Various types of support are spoken of, which are impacted by norms and preconceptions, and a silence and an absence of conversation about sexuality and sexual health is described.

#### 3.2.1 Taking initiatives for responsive conversations

The participants described the initiation of health-promoting conversations about sexuality and sexual health in a professional manner as being important. This includes taking the initiative to ask questions that are well suited, and being responsive to the patients’ needs. However, challenges are sometimes experienced, and the courage needed to take the initiative for conversations is lacking. A suitable approach to initiate such conversations about sexuality and sexual health was suggested as taking place in connection with a regular review of the patients’ medication, as side-effects can have a negative impact on sexuality. The participants spoke of needing to be responsive, for example, when patients request potency-enhancing drugs, and when they express a low or lack of desire. These issues are seen as related to the opportunity to relieve anxiety using masturbation, and participants speak of normalizing sexuality:

Address the problem with side-effects and the like, you may need to bring it up a little, a little bit and then pick it up again. You often have to split it up a bit into different small parts, but also to actually address that, that it is possible to have a normal sexuality, that it doesn’t have to be a problem, even if you’re locked up in forensic psychiatry (P3).

Furthermore, possible approaches for making sexuality and sexual health talkable are spoken of in connection with screenings and assessments that are carried out by various healthcare professionals. Examples are given of how occupational therapists have the possibility to initiate these conversations as they are used to having general conversations about sensitive and intimate topics. Using a team-based assessment instrument containing questions related to sexual health is also suggested as being helpful when asking follow-up questions that might otherwise have been overlooked. Examples are given of conversations regarding reproductive health and relationships, where the participants have had to be responsive. Recurring topics with patients are concerns about past and current relationships, but also about loneliness and longing for future relationships raising questions to the staff such as

How I’m ever going to be able to find someone when I’m in here in a secure unit, or How will I ever be able to find someone when I’ve been in a secure unit, no one will want to be with me? (P16).

When patients have concerns about gender identity and have gender dysphoria, the participants experience that they need to listen and let the patient describe what sexuality means to them. Furthermore, it is seen to be important to ask questions and talk about partner violence in close relationships, and to be responsive to whether the patient has had transactional sex. Participants speak of it being difficult to have responsive sexual health conversations with patients with severe mental illness, and examples of difficult areas are destructive relationships, destructive views of sexuality, unequal relationships, condescending attitudes towards women or that a patient has committed a sexual offence. It can be even more complex to work with patients who have intellectual disabilities, and who have been convicted of a sexual offence:

Then we expect them to behave differently, but without giving a single tool when it comes to sexuality and that part. They still have to understand the whole big thing, which is so difficult even for us who do not have an intellectual disability (P1).

#### 3.2.2 Dealing with norms and preconceptions

The participants describe that there can be a high threshold for conversations about sexuality and sexual health, as it can be perceived as private and taboo. It could also be a sensitive or charged topic in conversations among staff, sometimes making silence easier than discussions:

It’s just quiet about it, you, you just leave it out, until it becomes a problem. And then no one knows how to handle it and everyone, you easily fall into different morals. Opinion and much of one’s own morals and opinions and experiences are reflected in those discussions (P1).

According to the participants, different and individual norms and values influence the way patients are treated. Sexuality among patients who are heterosexual and have a gender expression that conforms to the norms are not discussed as much among staff, and they experience that these patients are subject to fewer reprisals. Gender also matters, as male patients who approach other patients sexually may be moved to a higher security class, while female patients are told to stop their behavior. Furthermore, women’s expressions of sexuality are reviewed more harshly, while their other actions in the wards are judged kinder. A more lenient treatment of female patients was motivated in terms of women more often being victims than perpetrators.

The participants describe that patients can be stigmatized in matters of sexual health and receive worse treatment, compared to people in general. Differences in treatment and support between patients (depending on diagnosis and ability to speak up for themselves) within forensic psychiatry are also mentioned;

No one did anything, it wasn’t addressed or discussed. Nor did he get an examination, he didn’t get any help, nothing, it was just swept under the carpet, yes, all the time, I experienced. I think it’s really disappointing (P14).

Patients could additionally be seen as non-sexual, and conversations between staff could sometimes evolve around patients having no need for a sexual life. The absence of systematic sexual health conversations left staff to depart from their own norms, values and preconceptions when educating patients about what is normal and not normal, and about which limits and regulations should apply. Staff could, for example, decide which books patients are allowed to borrow:

We have a book trolley that goes around the wards, and as recently as yesterday we were told that a ward had removed a book called The Wankers. The staff who had to take the book back felt that this was just a title of the book, the book itself was not controversial in any way. We shouldn’t limit our patients’ access to literature, but still, the ward thought that this isn’t appropriate, so they had to take it back, it shouldn’t be on a library trolley in the ward, mmm, I feel THAT’S how we often work (P5).

### 3.3 Being able, but also lacking knowledge and resources

In the third category, it is seen how the participants have varied levels of competence in terms of being able to converse about sexuality and sexual health with patients. A need for increased knowledge and resources is spoken of, as well as a desire for support that promotes initiatives for conversations about sexuality and sexual health.

#### 3.3.1 Lacking competence creates uncertainty

There is a wide range in terms of the level of competence among the participants; some say that they are confident in themselves, while others experience a lack of competence and feel insecure when having conversations regarding sexuality and sexual health with patients. Most had little or no education about sexuality and sexual health in their undergraduate education. There were participants who had attended lectures, e.g., about LGBTQI (lesbian, gay, bisexual, transgender, queer and intersex) issues provided at the workplace. Others had furthered their education or immersed themselves, e.g., into sexual side effects of psychotropic drugs, paraphilias, violence in intimate relationships or gender studies. The ones who felt they were competent describe that they, in addition to having conversations with patients, also train and supervise other staff about addressing issues about sexuality and sexual health.

Most participants claim that staff working in forensic psychiatry generally lack knowledge and competence concerning sexuality and sexual health. There was an evident uncertainty as they had similarly ambivalent emotions about having conversations with patients about what might be right and wrong to talk about. This uncertainty was related to a concern that patients could misinterpret the conversation and perhaps become (sexually) interested, or that (sexually) offensive behavior would be directed at staff. The participants’ concern of arousing something in the patient could lead to sexuality not being discussed;

One refrains from asking, because you are afraid of arousing something in your patient, but I think it’s already there, we just don’t talk about it (P10).

This fear could be related to participants experiencing a certain kind of challenge as they work around the clock with patients who are treated for long periods of time, sometimes many years. According to participants, their ability to address sexuality needs to be improved so that this uncertainty does not lead to silence:

Actually, I don’t know why we are so afraid to talk about it. Of course, it can be that you arouse or encourage things in the ward, and they shouldn’t have sexual relations with each other. If we have a permissive attitude maybe, and also have conversations about sexuality and sexual health, it might be interpreted that we have a permissive attitude. I don’t know if it that’s the case, or that it would increase the occurrence of relationships and vulnerability, I don’t know. I guess maybe that’s why we don’t talk about it (P5).

#### 3.3.2 Having knowledge and tools facilitates conversations

The participants experience that there is a lack of support and structure around conversations about sexuality and sexual health. It emerges that it is decisive which members of the staff are in the patient’s care team, whether or not the patient receives support, and, if so, what kind of support he/she receives. Conversations about sexuality and sexual health need to be included in a structured way in forensic psychiatric care, and supportive tools are suggested:

The right tools or that we feel safe in the conversations and know how to talk about it. Yes, I just think it’s important and an important topic of conversation to include (P11).

Suggestions are made that assessment instruments that are currently used by different healthcare professionals should be supplemented, or that additional tools could be developed. Furthermore, participants suggest that questions about sexuality and sexual health could be inserted into current routines such as on-going nursing assessments, annual overall follow-up in connection with the medication follow-ups, or as a part of the psychoeducational patient programs.

A permissive climate is described as one where the patient dares to seek advice. It is seen as equally important that both the patient and the member of staff feel safe to address and talk about sexuality, or as one participant says:

If we think it’s awkward, or if we think it’s hard, how can we ever believe that a patient will be able to open up about it? (P15).

The participants believe that a safe environment and an alliance between a patient and a member of staff can facilitate for patients to reflect upon themselves, their sexuality, relationships, and boundaries. In order to be able to provide this, the participants point out that it is important for them to have knowledge about how to refer the patients further when necessary, and to whom. Examples are given of how it is possible to refer to a nurse or physician regarding a medical problem; to a social worker or psychologist regarding problems with relationships; and to a physiotherapist if there is a need for increased body awareness. Moreover, a wish for a possibility to refer to a sexologist is mentioned.

The participants describe that they experience a need for increased knowledge and the development of an educational package containing general knowledge is suggested, e.g., about different sexual expressions and norms, as well as about the “how” of initiating and performing conversations. They highlight that all staff, regardless of profession, should receive the same educational package since this could facilitate inter-professional communication, and having practical exercises through workshops is suggested:

How you can talk about, sometimes we have had, yeah, but supervision, where you can roleplay yourself, so in this like how, in different conversations, about how to have, and mainly in substance abuse there has been these difficult conversations, that you get to practice about how to have these conversations. Perhaps in the same way here, through supervision or that you get to practice, that you get to practice with each other, to have these types of conversations (P11).

## 4 Discussion

We sought to explore how healthcare professionals experience talking about sexuality and sexual health with patients cared for in forensic psychiatry. The participants in this study needed to relate to and consider the dual mission in forensic psychiatry; to provide care and support the patients, but also to protect, which includes limiting the risks of exposure and protecting society. The findings showed that having these conversations involves a constant balance in relation to the dual mission, in the actual conversation with the patient, in considering norms and preconceptions, and in terms of the varying levels of knowledge among the staff and the resources available for them. Having to deal with this dual mission is, however, nothing new in forensic psychiatry, previous studies have shown the difficulty of balancing the dual roles (25–27). Those studies focus, however, on other topics; the gendered nature of health-promoting activities among nursing staff in forensic psychiatric care (25); patients’ views with regard to personal recovery in forensic psychiatry (26) and patient participation in a maximum security forensic psychiatric setting (27). These studies, together with our study, show that the dual mission pervades the care given in forensic psychiatric.

Several participants in the present study experienced that the mission to protect society is generally prioritized higher than the patients’ individual needs and rights for care. Nevertheless, the dual mission in forensic psychiatry care is described in a Swedish report as: firstly, to provide care to the patient and secondly, to protect the society from those patients who are at risk of recidivism (9). It is a complex assignment and due to the dual role of forensic psychiatric care in Sweden, ethical dilemmas are created (28). It is therefore a challenge to find a functional balance in the dual mission (26). Instead of walking balancing on a tightrope, the healthcare professionals struggle to maintain their balance on a slack line that sometimes sways.

This study revealed that the healthcare professionals have concerns whether the patient could misinterpret a situation. They describe a fear of arousing something, a fear that creates dilemmas for them when having conversations about sexuality and sexual health with patients, and in turn, which can lead to silence. Dein et al. (14) highlighted a similar dilemma of how patients’ sexuality is dealt with paradoxically in forensic psychiatry; healthcare professionals believed that patients who engage in sexual relationships are exposed to particular risks and this was dealt with by denying or negating their sexuality, and thus leaving these issuesunaddressed.

Having rules or prohibiting sexual relationships, may expose the patient to increased risks and be an obstacle in the development of future relationships (16, 19). It is thus important to have conversations about sexual health (13, 15, 16, 19). Such issues are an essential part of overall well-being, and it is important that the management of these issues is a part of the clinical care (18). This view is also shared by the healthcare professionals in this study when they maintained that it is important to initiate and open up for conversations about sexual health since it is a part of the patients’ general health.

An interesting finding was that the participants emphasized that the different healthcare professions, can have disparate views as to how the topic can be made more talkable and contribute to the field through, for example, screenings, assessments, and medical reviews. Other studies similarly emphasize that nurses have an important role in providing support to patients in forensic psychiatry and in providing sexual health education to help patients avoid exposure to sexual risks (16, 29). Addressing sexuality is also a domain for occupational therapists and it has been emphasized that sexuality is a part of occupational therapy, however, it is important to respect one’s own personal and professional boundaries and knowledge levels (4, 30). A so-called Occupational Perspective of Sexuality can support, not only occupational therapists, but all healthcare professionals, to address sexuality by using the concepts from the theoretical framework, the Occupational Perspective of Health by Wilcock and Hocking (31, 32); “*doing* sex(uality), *being* and *becoming* a sexual being and *belonging* to one’s sexuality” (31) (p.157).

The participants in the present study have a varied competence in terms of being able to address and converse about sexuality and sexual health with patients. The findings showed that when a lack of knowledge and competence prevails, an uncertainty is generated that makes it difficult to carry out health-promoting conversations about sexuality and sexual health. Similar findings are found in a study by occupational therapists where insufficient knowledge is a significant barrier to addressing sexual health and contributes to reduced comfort, confidence and competence (4). The fact that education and training on how to work with SRHR has only a very minor part in the Swedish higher education programs; e.g. in law, nursing, occupational therapy, physiotherapy, psychology, social work, and undergraduate medicine, may lead to healthcare professionals being uncomfortable when discussing sexual health issues with their patients (33).

In addition to a lack of knowledge, the result indicates that initiating responsive conversations about sexuality and sexual health is also a challenge since it is a field fraught with norms and preconceptions. The participants in the present study describe it as being important to focus on health-promotion and responsive conversations with patients in a professional manner, at the same time, this is experienced as something difficult and taboo. The example, raised by one participant, concerning the removal of a book because of its title, judging the book by its cover, is an example of the prevailing preconceptions. Another problematic behavior found in this study, motivated by the reduction of the risk of exposure to other patients, is that the patients were sometimes hindered from choosing gender-crossing clothing. Dein et al. (14) have described that restrictions on sexual expression may lead to ending existing relationships, making it difficult to make new relationships, and even damaging the patient’s identity as a sexual being. Further, not being allowed to freely express one’s own gender or sexuality is not in line with the sexual rights (1, 3). Achieving sexual and reproductive health within forensic psychiatry thus requires the recognition of sexual and reproductive rights, based on human rights.

The participants in this study emphasized that having knowledge and tools could facilitate conversations. They suggested that assessment instruments should be supplemented, or additional tools developed. This is in line with previous findings that suggest that reviews of sexual health should be a standardized part and that healthcare professionals should provide support to patients in forensic psychiatry through sexual education programs (18). Based on our findings and those in previous studies, psychoeducation and relationship counseling should be included in strategies to treat antipsychotic-induced sexual dysfunction (17), and sex education ought to be made routinely available and made as part of rehabilitation care for all patients within forensic psychiatry (18, 19).

The participants also spoke of the need of a permissive climate for both the patients and the staff to feel safe having conversations about sexuality. Education is important in order to be able to change attitudes, and it has been concluded that there is a need to reduce stigma around mental health in general and about sexuality in particular, including LGBT issues (14, 29). Brand et al. (18) claim that it is necessary to have a professional understanding of sexuality and sexual activity in patients with serious mental illness, in order to identify their support needs. Although it is highly relevant to discuss sexual problems, there is a reluctance, among both patients and staff, to do so (17). Regardless of the reluctance, the staff need to feel secure in conversations, and refrain from passing on feelings of judgment or shame, so that patients dare to raise their sexual health problems (18).

### 4.1 Strengths and weaknesses

The result in a study with a qualitative design are not generalized and instead trustworthiness of the result needs to be taken into consideration in order to evaluate the strengths and weaknesses of the study. This was performed using the concepts of credibility, dependability and transferability (21). Credibility and dependability were sought through motivating and describing the study design, selection of participants, data collection and analysis in as detailed a manner as possible. A varied sample was achieved in terms of profession, gender, age, and work experience, which is seen as a strength. A further strength is the researcher collaboration and triangulation during the analysis, where each author’s perspectives and experiences were useful. A weakness is that the transferability of the findings is limited to the local Swedish context. However, the findings can be of interest to others.

### 4.2 Clinical implications and future research

The study indicates that there is a lack of support and structure around conversations about sexuality and sexual health in forensic psychiatry care. Increased knowledge among healthcare professionals, as well as opportunities to practice the “how-to-initiate” conversations about sexual health with patients could make staff more comfortable. Staff being comfortable in discussing matters related to sexuality are hypothesized to be more competent in both promoting patients’ sexual health and preventing unwanted sexual approaches aimed at themselves or at other patients. Sexual health can be an important part to address in preparation for being part of society and ‘everyday life’ again, as sexuality is a part being human through life and maintaining one’s own identity. A plausible approach, which can make sexuality and sexual health talkable, is to address these issues in connection with different screenings and assessments that are already carried out by healthcare professionals. To support staff, the forensic psychiatric services need to acknowledge the national guidelines for SRHR, and the local guidelines need to be developed and adapted. Local interprofessional dialogues between healthcare professionals is recommended in order to gain greater clarity concerning who can do what, and which profession can be referred to regarding which issues. Further dialogue is also recommended about how healthcare professionals can get support in terms of managing the ethical dilemmas that can arise, in order to reduce ethical stress and promote a good working environment.

The participants in this study experience that patients have knowledge gaps concerning sexuality and sexual health, both factual and emotional knowledge. There is limited research available on forensic patients’ sexual development, experiences, health and knowledge (13). However, a recent study, carried out in Australia, showed that patients had reasonable knowledge of physiology, intercourse and sexuality, but less about pregnancy, contraception and sexually transmitted infections (34). Despite the patients having extensive contact with the forensic psychiatric services, only twelve percent had received any sexual education from healthcare professionals (34). The present study dealt with professional’s views on conversations about sexuality and sexual health. Further knowledge is warranted, focusing on the views of patients in the context of forensic psychiatry in Sweden.

### 4.3 Conclusion

The experience of balancing the dual mission leads to tension within the field of sexuality and sexual health that is difficult to navigate for the healthcare professionals, i.e. promoting health for the patients, and at the same time they need to limit risks of exposure by protecting patients, as well as society. The findings in the study form the overarching theme, which illustrates that talking about sexuality and sexual health with patients in forensic psychiatry was experienced as “Balancing on a slack line”, which in comparison to balancing on a taut line is experienced as being more demanding and challenging. Patients in forensic psychiatry belong to a group who are extra vulnerable and who are detained and cared for under coercion and addressing their sexuality and sexual health is experienced as important, but also demanding and challenging for healthcare professionals.

Further knowledge and resources for healthcare professionals are needed in order to be able to balance the dual mission related to conversations about sexual health. Forensic psychiatric services need to acknowledge the national guidelines on sexual and reproductive health and rights and provide education and support for healthcare professionals, so that they do not experience these conversations as though they are balancing on a swaying slack line. A balance in the dual mission, between sexual health-promotion and protection of society, needs to be found, which can be achieved through further dialogue within the healthcare team. In summary, there is a need to talk about sexuality and sexual health, provide support for the patient in terms of SRHR, and in order to better meet the patients’ need for support, further efforts both within the clinic and in research are required.

## Data Availability

The datasets presented in this article are not readily available due to privacy restrictions. Requests to access the datasets should be directed to AL, anna.lindskog@gu.se.
